# A mixed-methods study of problematic social media use, attention dysregulation, and social media use motives

**DOI:** 10.1007/s12144-022-03472-6

**Published:** 2022-08-08

**Authors:** David Caelum Arness, Theodora Ollis

**Affiliations:** School of Psychology, Western Sydney University, Sydney, NSW Australia

**Keywords:** Problematic social media use (PSMU), Social media addiction, Attentional dysregulation, Self-regulation, Social media use motives

## Abstract

Problematic social media use (PSMU) refers to excessive uncontrolled use of social media which impacts upon daily functioning (Blackwell et al., 2017). Self-regulation is central to the development and experience of PSMU, and conceptually interrelates with individual usage motivations (Reinecke et al., 2022). While there is a growing body of research on social media use motivations, how usage motivations and self-regulation combined influence PSMU is not well understood. There are also persistent questions around the effectiveness of addiction-based measures of PSMU. The quantitative component of this nested mixed-methods study (N = 607) employed hierarchical regression and structural equation modelling, principally identifying that impulsive social media usage mediates the pathway between perceived executive/attentional functioning and the Bergen Social Media Addiction Scale (BSMAS, Andreassen et al., 2012, 2016), a popular tool used to measure PSMU. In contrast, social-engagement motivations had a negative influence on the BSMAS. The qualitative component, comprising interview/open-ended questionnaire, explored individual experiences self-regulating social media use. Participants (N = 24) were recruited from the survey study, based on meeting screening criteria for executive dysfunction (Adult Self-Report ADHD Scale, Kessler et al., 2005), with sub-groups defined by top and bottom quartile BSMAS scores (evenly grouped). Thematic analysis found that most individuals with attention dysregulation, regardless of their BSMAS category, perceive self-regulation of social media use as highly challenging and effortful, describing broadly problematic relationship with social media. They also described rich combination of motivations and context of using social media, and strategies for managing use. This research questions the effectiveness of the BSMAS as a measure of general PSMU (lacking a formed self-regulation component), especially in individuals with attentional dysregulation. Future research investigating self-regulation strategies and focusing on characteristics of positive social media use is needed.

## Introduction

Over the past two decades, social media use has become an increasingly popular way to stay in contact with friends and family, find entertainment and pass time. Social media refers to internet-based sites and applications on which users can create public or semi-public profiles, interact with others, and share user-generated content (Boyd & Ellison, 2007; Paakkari et al., 2021). Approximately 90% of emerging adults, aged 18 to 29 years, report using social media daily, and a majority have active accounts on at least five different social media platforms (Scott et al., 2017). Furthermore, the global average time spent per day engaging with social media is increasing steadily, from a reported 1.5 h in 2012 to almost 2.5 h in 2020 (Tankovska, 2021). In a recent study by Schivinski and colleagues (2020), over 96% of participants reported accessing social media via a smartphone, and the most frequently used social media platforms in order were: Facebook, Instagram, Twitter and Snapchat.

Social media helps to form and maintain social connections, and to create supportive communities for individuals from diverse groups, including LGBT + adolescents, individuals from ethnic minorities and those with chronic illnesses (Shapiro & Margolin, 2014; Van Den Eijnden et al., 2018). A qualitative study by Radovic and colleagues (2017), which explored the positive and negative uses of social media in adolescents with depression, reported that some depressed teens use social media to seek encouragement and inspiration or for self-reflection via a private online journal. Further benefits of social media use include increased perceived social support and decreased loneliness (Best et al., 2014; Lee et al., 2013). However, despite many benefits of social media, popular narrative often focuses on the negative outcomes for users, which is reflected in the research literature (Owenz, n.d.).

## Problematic Social Media Use

Problematic social media use (PSMU) refers to the excessive, uncontrolled use of social media platforms that leads to detrimental effects on the users’ functioning and wellbeing (Kuss & Griffiths, 2017; Blackwell et al., 2017). Schivinski and colleagues (2020) reported PSMU at a prevalence of 6.68% of social media users, while other studies have estimated that 7–11% of adolescents are problematic users (van den Eijnden et al., 2016; Eijnden et al., 2018). In their recent study, Paakkari and colleagues (2021) reported an additional 33.5% of social media users as at moderate risk of developing problematic use of social media, a large proportion of whom were young women with low parental monitoring. Paakkari et al. further report health and wellbeing impacts associated with PSMU, including headaches, neck pain, shortened sleep, irritability, nervousness, and loneliness. Similarly, previous research has documented links between PSMU and indicators of mental health such as anxiety, depressive symptoms, and lower self-esteem (Andreassen, 2015), decreased psychological wellbeing and life satisfaction (van den Eijnden et al., 2018; Wang et al., 2016), and poorer outcomes in academic study, as it was found to be a significant predictor of lower GPA (van den Eijnden et al., 2018) and academic procrastination (Yildiz Durak, 2020; Lian et al., 2018) in high school and undergraduate students respectively. Furthermore, a systematic review by Kokka and colleagues (2021) found that problematic internet use is associated with disrupted sleep patterns, shortened sleep time and poorer sleep quality. The severity of problematic internet use was also found to be negatively related to psychological wellbeing (Mei et al., 2016) and is linked with depressive symptoms and feelings of loneliness (Vigna-Taglianti et al., 2017).

However, there is a growing call for researchers to adopt a more cautious and critical approach to PSMU, noting similar patterns of research on negative impacts (followed by more conservative revision) in other areas of new technology use, such as with internet use and online gaming (Ellis, 2019; Aarseth et al., 2017). Of particular impact, Orben and Prybylski (2019) argue that many of the strong claims about the negative impacts of technology use are driven by flawed assumptions that large-scale empirical data ensures robust conclusions. They evaluated the impact of technology use (including social media use) on adolescent psychological wellbeing, sourcing data from three large national longitudinal health and wellbeing surveys from the US and UK. The authors employed Specification Curve Analysis, a statistical approach that minimizes the impact of researcher decisions on the selection of variables and their relationships for analysis, especially problematic in large cohort data with many variables where small effects can generate significant results. Orben and Prybylski report that while social media use had a negative association with wellbeing, the effect was small, accounting for less than 0.1% of the variability in wellbeing. They further contextualize the findings by noting that the overall impact of technology use on wellbeing was substantially less than the impacts on wellbeing associated with other common adolescent experiences, such as being bullied and binge drinking, and ranked somewhat more closely to the association for eating potatoes. While sobering, the authors note that issues with the measures of technology use limit the generalisability of the data. This is particularly true of social media use, which is measured in terms of self-report hours of usage.

Time spent on social media may correlate with PSMU as problematic users in general are probably more likely to spend time on social media, but there is much variance in simple usage characteristics. For instance, highly engaged productive activities like hosting a YouTube channel would naturally result in much of one’s time being spent on and thinking about social media, but this would be a very poor indicator of whether such an individual’s use was problematic or not. This is borne out in the research, with recent meta-analysis showing that “screen time”, including time spent on social media specifically, was unrelated to mental health outcomes (Ferguson et al., 2022). In research on PSMU, Yildiz Durak (2020) found no statistically significant relationship between Social Media Disorder Scale (van den Eijnden et al., 2016) and the duration of time spent on social media per day in a sample of 451 Turkish high school students. Similar results are reported by Boer et al. (2021) in an investigation involving 2,109 Dutch high school students. Indeed, van den Eijnden and colleagues (2018) distinguish between heavy social media use and addictive social media use. They found positive social outcomes, including the formation and development of friendships, for individuals in the heavy social media use group, compared with decreased psychological well-being and life satisfaction for individuals in the addicted social media use group.

It is noteworthy that much of the research on PSMU is presented in or traces back to an addiction (or use disorder) framework. Popular measures, such as the Bergen Social Media Addiction Scale (BSMAS; Andreassen et al., 2016) and the Social Media Disorder Scale (SMDS; van den Eijnden et al., 2016) correspond to diagnostic themes of addiction: salience, craving, mood modification, escape, withdrawal, and conflict (Andreassen et al., 2016). While social media addiction (SMA) has gained traction and is a fast-growing research area (Sun and Zhang, 2021), there are important limitations that are not routinely considered in research on PSMU/SMA. Conceptually, as with internet and smartphone use, social media use involves a wide range of possible behaviours and activities, and it is not clear how these various kinds of behaviours articulate with general measures of SMA (Lee et al., 2017; Carbonell & Panova, 2017).

Despite the wealth of research on PSMU/SMA, much of it is considered as being at the stage of initial screening research, being correlational, cross-sectional, self-report studies focusing on young adult university students (Carbonell & Panova, 2017; Ellis, 2019). Carbonell & Panova further highlight that research using such screening tools (e.g., the BSMAS) are fraught with false positives, as they normally do not indicate specific behaviours that can be used for validity testing, and there is no clinical definition that can be used as a gold standard. Moreover, there are concerns with survey-based addiction screening tools, which tend to have low predictive value, especially where the prevalence of a particular disorder is low (Maraz et al., 2015), and as such, the tools should only be used as an early detection mechanism, not to draw conclusions around the nature of the construct or to identify whether certain behaviours are pathological. Also, and perhaps most importantly, the addiction framework has much potential to problematize normal behaviour, without necessarily predicting problematic behaviours or outcomes (see Aarseth et al. 2016 for a discussion of this related to gaming, and Ellis 2019 in relation to smartphone use, and Carbonell & Panova for similar discussion of social media).

### Importance of self-regulation

Whilst there are some deep challenges in the area of SMA, and consequently within PSMU, there is a growing recognition of the centrality of self-regulation to understanding PSMU, echoing similar trends in other areas of human-technology interaction, such as smartphone use (Busch & McCarthy, 2021), and internet use (Kumar Sinha et al., 2020; Mei et al., 2016). In a study of adolescent PSMU, self-regulation was negatively related to PSMU, such that adolescents who were able to regulate their social media use had a decreased likelihood of developing PSMU (Yildiz Durak, 2020). Indeed, Reinecke et al. (2022) argue that self-regulation is a key boundary condition, distinguishing between problematic and non-problematic use.

When considering the role of self-regulation, the context of attention deficit hyperactivity disorder (ADHD) presents as an interesting focus as it is clinically characterised by difficulties with self-regulation (inattention, hyperactivity, and impulsivity; American Psychiatric Association, 2013). It is important to recognise that the experience of ADHD (indeed neurodiversity and other differences generally), should not be discussed solely in terms of the limitations imposed by a deficit model (Dinishak, 2016). The environments within which most people with ADHD are required to function are often not conducive to self-regulation, however, and so the challenges to self-regulation (and capacities thereof) tend to be highly salient. Unsurprisingly, ADHD has been positively correlated with PSMU (Andreassen et al., 2016; Hussain & Griffiths, 2021; Merelle et al., 2017; Ra et al., 2018), and with related areas of problematic internet and smartphone use (Cakmak & Gul, 2018; Demirtaş et al., 2020; Evren et al., 2018; Panagiotidi & Overton, 2020; Wang et al., 2017). In a recent longitudinal study investigating ADHD symptoms and social media use, Boer and colleagues (2020) found greater social media use intensity and social media use problems to be correlated with greater ADHD symptoms in a sample of 543 Dutch adolescents. Similar findings were reported by Ra et al. in a diary study, and while not focused on ADHD, Du et al. (2021) tracked 329 adult users of social media over 4 months and identified a reciprocal relationship between mindful awareness (which reflects processes of self-regulation) and self-control failure, such that self-control failures impaired mindful awareness, which in turn increased future self-control failures.

Though more research is required, self-regulation is clearly important for individual control of social media use, and it has been suggested that subclinical ADHD inattention symptoms contribute to PSMU and problematic internet use (Panagiotidi & Overton, 2020). Lee and colleagues (2021) explored the relationship between inattention and PSMU in their study of functional connectivity differences between problematic and non-problematic social media users within the dorsal attention network and dorsolateral prefrontal cortex (DLPFC). The dorsal attention network is responsible for top-down control of attention and the DLPFC is suggested to be involved in executive control (Ceranoglu, 2018; Lee et al., 2021). Lee and colleagues (2021) found that problematic users of social media had weaker functional connectivity between these two regions, indicating deficits in prefrontal attention control, which contribute to poor self-regulation of social media use. Whilst the cause of failures in self-regulation are complex, individual hedonic state and tolerance to boredom may be an important mechanism. Indeed, there is an unsurprising link between PSMU and boredom proneness (Stockdale & Coyne, 2020), which has also been shown in the context of problematic smartphone use (Elhai et al., 2018), with Stockdale and Coyne also noting that it is a trait characteristic of ADHD.

### Social Media Use Motives

Motivations for using social media, such as the alleviation of boredom or for procrastination, have been found to be associated with PSMU, social and psychological outcomes, and characteristics of social media use (Korhan & Ersoy, 2016; Meier, 2022; Omar & Dequan, 2020; Schivinski et al., 2020). Uses and gratifications theory, developed in communications research for media such as radio and newspaper and naturally extended to new media, considers the individual’s active choice to engage with media to satisfy their social and psychological needs (Dolan et al., 2016; Whiting & Williams, 2013). In essence, individuals will engage with social media to the extent that features and affordances fulfil their needs and desires (Liu and colleagues, 2020). Social media use motives vary between studies but typically include some form of the following: (a) information seeking, (b) social connection, (c) entertainment, (d) escapism, (e) self-expression and (f) surveillance (Dolan et al., 2016; Korhan & Ersoy, 2016; Omar & Dequan, 2020; Schivinski et al., 2020; Süral et al., 2019; Wang et al., 2016; Whiting & Williams, 2013).

Social media use motives have been associated with a variety of outcomes related to PSMU, including psychological distress, depression, and anxiety (Stockdale & Coyne, 2020). For example, Rae & Lonborg (2015) found that Facebook users who used the social media platform primarily to form new social connections had significantly higher levels of depression, anxiety and loss of behavioural/emotional control, than those who use social media to maintain existing relationships. In contrast, using social media with the motivation of seeking information is not associated with negative outcomes in mental health or behaviour (Stockdale & Coyne, 2020). Moreover, self-expression and passing time have been found to be related to excessive use of social media, both for individuals who engage in excessive use of Weibo and those who do not (Wang et al., 2016). Similarly, Süral and colleagues (2019) found that self-presentation and escapism may lead to PSMU.

Given that motives and characteristics of social media use define how we engage with social media, such motives may provide a link to understanding the development of PSMU. Indeed, a growing body of research demonstrates various roles that use motives have on productive and problematic use. However, there is a gap in understanding the role of self-regulation in the interplay of social media motives and PSMU. The present study uses a nested mixed methods design to: (1) investigate the relationship between PSMU, attention dysregulation, and social media use motives (entertainment, information seeking, social interaction, procrastination, stress relief, and unintentional/habitual use), along with indicators of psychological wellbeing (anxiety, depression, and stress); and (2) explore the experiences of social media use in university students with indicators of attention dysregulation. We expect that attention dysregulation will be a significant predictor of PSMU, and that both attention dysregulation and PSMU will be positively related to social media use motives of procrastination, and unintentional habitual use, while purposeful adaptive use motives (social, information seeking, entertainment, and stress relief) may reduce PSMU. The ambiguous role of wellbeing in PSMU makes it unclear what role it may take. The qualitative component will explore the perspectives and experiences of participants in view of their ability to self-regulate social media use and their motivations to use social media.

## Methods

The current study implemented a nested mixed methods design, allowing for deeper investigation into attention dysregulation and social media use motives as related to PSMU. Quantitative methods were Structural Equation Modelling, guided by Exploratory Factor Analysis and hierarchical regression, to investigate the influence of attention dysregulation and social media use motives on PSMU. Interviews and open-ended questionnaires were used to explore the experiences of individuals with indicators of attention dysregulation when self-regulating social media use and their motivations to use social media. Ethics approval was obtained through the Western Sydney University Human Research Ethics Committee prior to commencement of the study.

### Participants

Participants were recruited from a pool of first-year psychology students through the Western Sydney University SONA portal. Participants were incentivised to complete the 30-minute survey with a course credit reward. Informed consent was gained from participants prior to commencing the survey. Within the survey, participants were asked about their social media use and completed self-report measures of ADHD symptoms, psychological wellbeing (depression, anxiety, and stress), PSMU, and social media motivations.

The interview pool was selected based upon two criteria: (1) a clinical ASRS score of 5 or greater, and (2) a score within the highest or lowest quartile on the BSMAS, to ensure a range of social media use experiences in the sample. Participants who met these criteria were emailed an invitation to complete an interview or an equivalent open-ended questionnaire. Interviews were conducted via video call and lasted on average 20 min. Prior to the interview, participants consented to being recorded and for their responses to be used in the study. Interview recordings were transcribed for thematic analysis. Alternatively, participants could opt to complete an open-ended questionnaire on Qualtrics in the place of an interview. Participation in the interview or open-ended questionnaire was incentivised with further course credit.

#### Participants for the quantitative study

A total of 703 first-year psychology students, recruited from amongst the 2021 Autumn and Spring cohorts at Western Sydney University. Following data screening, six participants were omitted from the dataset as multivariate outliers, while an additional 90 were flagged as low effort responders. Therefore, 607 participants were included in this study, ranging in age from 17 to 58 (M = 22.02, SD = 6.179). The sample was made up predominantly of individuals identifying as cisgender women (475 women, 126 men, 4 non-binary, 2 undisclosed).

#### Participants for the qualitative study

From the initial sample, 24 participants between ages 18 and 31 (19 women, 5 men, M = 20.8, SD = 3.9) volunteered to join the qualitative study, completing either an interview (n = 7) or equivalent open-ended questionnaire (n = 17).

### Measures

#### Quantitative study

##### Bergen Social Media Addiction Scale (BSMAS)

The BSMAS (Andreassen et al., 2012, 2016) was used as a measure of PSMU as it is a popular, brief (6-item) measure. It is scored on a five-point scale from 1 (very rarely) to 5 (very often), framing six features of purportedly addictive use: salience, tolerance, mood modification, relapse, withdrawal, and conflict. We adopted the more common total sum scoring approach, rather than dichotomous cut-off scoring. The BSMAS is a widely used measure within PSMU research with research claiming validity (e.g., Balcerowska et al., 2022), however, there is some evidence of structural issues, such as Watson et al. (2020) indicating problematic fit and an (unspecified) item failing to load with the others.

##### Adult ADHD Self-Report Scale (ASRS)

The ASRS (Kessler et al., 2005) was used as a measure of attention dysregulation via ADHD symptoms. This measure has been used in both clinical and research settings as a screening tool for ADHD and has been found to have high validity and accuracy. It is a six-item measure in which participants respond to questions about how frequently they experienced the given symptom in the past six months. Responses are given on a five-point scale from 1 (never) to 5 (very often) and scores are summed to form a total score. A clinical cut-off is assigned to each item and participants are scored either 0 (doesn’t meet cut-off) or 1 (meets cut-off). These scores are summed to form a clinical ASRS score. The authors present evidence for convergent and discriminant validity, and test-retest reliability has been demonstrated in non-ADHD samples (Silverstein et al., 2018).

##### Social Media Motivations

Participants completed an ad-hoc 12-item measure of social media use motives, responding on a five-point scale how closely their motivations to use social media aligned with given statements, from 1 (not at all) to 5 (exactly). An average for each use motive is then calculated. Informed by research in the use and gratification area (e.g., Whiting & Williams 2013, who reported categories of uses most cited by respondents), we developed items assessing the following use characteristics: entertainment (e.g., I use social media because it is fun); social interaction (e.g., I sue social media to engage with other people); information seeking (e.g., I use social media for study/research); stress management (“I use social media to deal with stress”); procrastination (e.g., “I use social media even though I have more important things to do”); and habit (e.g., “I start using social media before I consciously realise I’m doing it”). Two questions are aligned with each category. While other researchers have used more extensive measures of use and gratification motivations (see for instance Lee & Kim 2014, who delve into uses such as surveillance and network expansion), we focus on broader motivational categories or characteristics of usage.

##### Depression Anxiety Stress Scale − 21 (DASS-21)

The DASS-21 (Lovibond & Lovibond, 1995) was used as a measure of depression and anxiety symptoms. The DASS-21 is a frequently used measure in both clinical and research settings and has demonstrated excellent validity and internal consistency (Antony et al., 1998). It is a 21-item measure composed of three subscales: depression (ɑ = 0.97), anxiety (ɑ = 0.92), and stress (ɑ = 0.95). Participants respond on a five-point scale from 1 (does not apply to me at all) to 5 (applied to me very much, or most of the time) to statements about how they have felt in the last week. Scores are then summed to form three total scores, one for each subscale. Psychometric qualities of this scale have been established, with adequate construct validity in non-clinical samples (Henry & Crawford, 2005).

#### Qualitative study

Participants completed either a semi-structured interview via video call or an open-ended questionnaire through Qualtrics which explored their experiences with social media use. Participants responded to questions about: (a) their social media use, (b) their motivations to use social media, (c) the impact of social media use on their lives, and (d) their experiences of self- regulating or reducing social media use. A copy of the open-ended questionnaire is included in Appendix A, the live interviews followed the same protocol and included the same prompts, but with differences naturally emerging depending on individual conversational dynamics.

### Data Analysis

#### Quantitative study – initial screening

The survey data was screened for outliers and violations of parametric assumptions using SPSS (IBM SPSS Version 25.0, 2017). Univariate analysis found no abnormalities and no participants were omitted from the dataset as univariate outliers. The dataset was screened for multivariate outliers using Mahalanobis distance and six participants were omitted as multivariate outliers. Additionally, adapting recommendations in the psychometric literature (Meade & Craig, 2012; Niessen et al., 2016), 90 participants that displayed evidence of insufficient effort responding were flagged as potentially unreliable data; this included those participants who spent an improbably low time on the survey (< 10 min, based on initial pre-testing), and those who demonstrated low odd/even test reliability. Removing these participants did not change the overall outcomes, but did improve model strength, and so this is the data reported. The parametric assumptions of multiple regression were satisfactory, which offers initial confidence for SEM. The assumptions of normality, linearity and homoscedasticity were checked through a scatterplot of residuals and predicted values. There was no evidence of multicollinearity with acceptable VIF scores and no correlation between predictor variables > 0.9.

#### Qualitative study - approach

Interview transcripts and written responses from open-ended questionnaires were screened and cleaned, then analysed according to Braun and Clarke’s (2006) six steps of thematic analysis. Broadly, this involved familiarisation with the dataset, initial coding, refining of codes, grouping of concepts and finally forming themes. An inductive approach was utilised in this study, with themes developed from the data. This process produced two themes and seven subthemes.

## Results

### Quantitative results

A core aim of this research is to investigate the relationship between PSMU, attention dysregulation, and social media use motives (entertainment, information seeking, social interaction, procrastination, stress relief, and unintentional habitual use). We expect that individual characteristics of social media usage will differentially influence PSMU, in particular: exacerbated by impulsive characteristics, and mitigated by engaged and social usage. A Structural Equation Modelling (SEM) approach would provide a powerful tool to investigate these relationships, as long as the data satisfies SEM requirements. It was deemed prudent to commence with an Exploratory Factor Analysis (EFA), however given the amount of detail, this is presented in Appendix B. Correlations between factors developed from the process are presented in Table 1.

**Table 1:** Pearson Correlations for Social Media Use Motives and Key Outcome Measures (BSMAS and ASRS)

	Variable	1.	2.	3.	4.	5.	6.	7.
1.	BSMAS	—						
2.	ASRS	0.64	—					
3.	DASS_A	0.40	0.58	—				
4.	DASS_D	0.43	0.61	0.88	—			
5.	Impulsive use	0.78	0.67	0.34	0.36	—		
6.	Social use	0.18	.03^a^	0.09*	− 0.01 ^a^	0.38	—	
7.	Engaged use	0.60	0.33	0.28	0.25	0.80	0.65	—

BSMAS = Bergen Social Media Addiction Scale; ASRS = Adult ADHD Self-Report Scale; DASS_A is the DASS depression scale; DASS_A is the DASS anxiety scale

^a^ NSIG

* p < .05 (all other *p* < .001)

This data shows strong correlations between all variables and the BSMAS, but especially for impulsive use, engaged use, and attentional functioning (recall that as an ADHD screening tool, high scores on this measure indicate increasing attentional dysfunction). Social use has the weakest correlation with the BSMAS, albeit still significant, and further shows the weakest correlation with most other variables. To gain an initial sense for modelling these variables, a hierarchical multiple regression was conducted with BSMAS as the criterion, presented in Table 2. Notably, attention dysregulation maintains a positive predictive effect on BSMAS, while impulsive use emerges as the strongest individual predictor. The reduction in coefficient strength for attention dysregulation with the addition of usage characteristics (from *β* = 0.606, *p* < .001 to *β* = 0.105, *p* = .013) suggests a potential mediating relationship (most likely from impulsive use *β* = 0.715). Engaged entertainment use (i.e., motivations to use for entertainment, information seeking, and as a way to relieve stress) does not predict BSMAS (*β* = − 0.029, *p* = .466), while social connection motivation shows a significant negative relationship (*β* = − 0.081, *p* = .011). Neither anxiety nor depression predict PSMU (*p* > .1 for both)

**Table 2:** Hierarchical Regression Results Predicting PSMU (BSMAS) from Attention Dysregulation (ASRS), Anxiety, Depression (DASS-21), and Categories of Social Media Use Motives

Model		Unstandardized Coefficients		Std. Coefficients	t	Sig.	R^2^
		B	Std. Error	Beta			
1	(Constant)	0.830	0.060		13.855	< 0.001	0.415***
	Attention	0.639	0.031	0.645	20.735	< 0.001	
2	(Constant)	0.815	0.062		13.060	< 0.001	.418^a^
	Attention	0.601	0.039	0.606	15.385	< 0.001	
	Anxiety	− 0.026	0.072	− 0.024	− 0.366	0.714	
	Depression	0.068	0.053	0.087	1.300	0.194	
3	(Constant)	0.114	0.081		1.404	0.161	0.648***
	Attention	0.104	0.042	0.105	2.488	0.013	
	Anxiety	0.057	0.058	0.052	0.984	0.326	
	Depression	0.056	0.042	0.071	1.331	0.184	
	Social	− 0.055	0.022	− 0.081	-2.547	0.011	
	Engaged	− 0.027	0.037	− 0.029	− 0.729	0.466	
	Impulsive	0.468	0.031	0.715	15.199	< 0.001	

^a^ NSIG F-change

*** p < .001

#### Post-hoc research questions

**Fig. 1:**
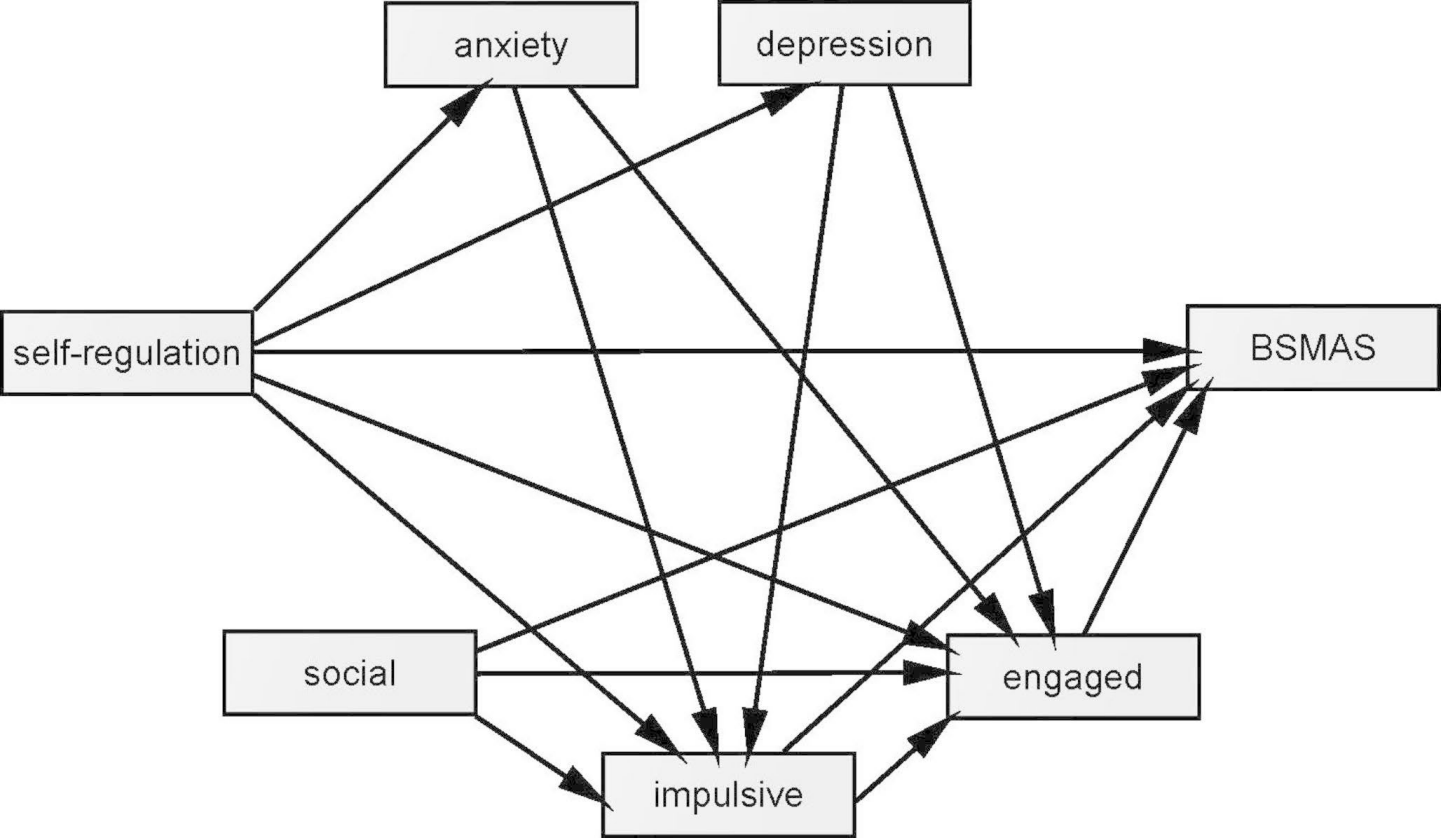
Path diagram with hypothesised path structure, predicting PSMU (BSMAS) from attention dysregulation (ASRS), mediated by social media use motives (social, engaged, and impulsive), and wellbeing factors (DASS anxiety and depression).

From the above data exploration, SEM is used to evaluate predicted relationships between attentional dysregulation, social media use motives, and PSMU. This evaluation is represented by the structural diagram (Fig. 1), with 3 guiding questions:

1. Does attention dysregulation (measured by ASRS) impact PSMU (measured by BSMAS), and is this relationship mediated by impulsive, engaged, and/or social-connection oriented social media use?
2. Given that motivations oriented to social connection were uncorrelated with attention dysregulation, and low to zero correlation with anxiety and depression, we investigate whether social motivations influence PSMU separately, and whether this influence is mediated by impulsive and/or engaged usage.
3. Finally, we examine whether wellbeing indicators (DASS anxiety and depression) mediate the relationship between attention function and PSMU, through either impulsive or engaged use.

#### Confirmatory factor analysis and structural equation Model

AMOS (version 28) was used to construct a Confirmatory Factor Analysis (CFA) and test the measurement model. An initial fully covaried model demonstrated good fit, with χ^2^/df = 2.456, Goodness of Fit Index (GFI) = 0.881, Tucker Lewis Index (TLI) = 0.920, Root Mean Square of Error Approximation (RMSEA) = 0.049, and Hoelter Critical Number (CN) = 271.

Reliability was strong for all factors, with Max Reliability (H) > 0.7 for each. However, some issues with construct validity were observed. Following Fornell and Larcker (1981), and Hair et al. (2010), convergent validity was problematic, with Average Variance Extracted (AVE) below the 0.5 threshold, for BSMAS (0.46), ASRS (0.38) and engaged usage motivation (0.47), while issues with discriminant validity were observed for DASS anxiety and BSMAS factors with Maximum Shared squared Variance (MSV) higher than AVE. Removing low-loading items (2 from BSMAS, 1 from engaged use, 2 from ASRS, and 1 from DASS anxiety) largely resolved these validity issues: convergent validity becoming adequate for engaged use, and very close to the 0.5 AVE threshold for BSMAS (0.499) and for ASRS (0.486), with discriminant validity issues persisting only for anxiety. However, the model derived after adjusting factors did not alter the SEM outcomes, and so we retained the original items to maintain conceptual meaning of those factors. However, due to the noted validity concerns, we urge caution with interpreting the data, and emphasise that replication with alternate measurement tools and sample groups is required.

Common Method Variance (CMV) is known to be both a measurement and design issue (Podsakoff et al. 2003; Spector 2006), especially present in cross-sectional surveys (Jordan & Troth, 2020). Our survey design aimed to guard against aspects of respondent bias by ensuring anonymity, using varied scale properties, and breaking the survey into sections. Data screening to remove low effort responders (i.e., long string and elapsed time) and multivariate outliers further enhanced these efforts. We confirmed no significant CMV using Lindell and Whitney’s (2001) approach, selecting a marker variable (the describing subscale of the Five Factor Mindfulness Questionnaire, Baer et al., 2006) according to recommendations of Simmering et al. (2015). Please see Appendix C for more detail on this process. All zero-order correlations between predictor and criterion variables remained significant after adjusting for CMV, and therefore are not likely accounted for by CMV. Given this, composite variables were imputed without adjustment.

**Fig. 2:**
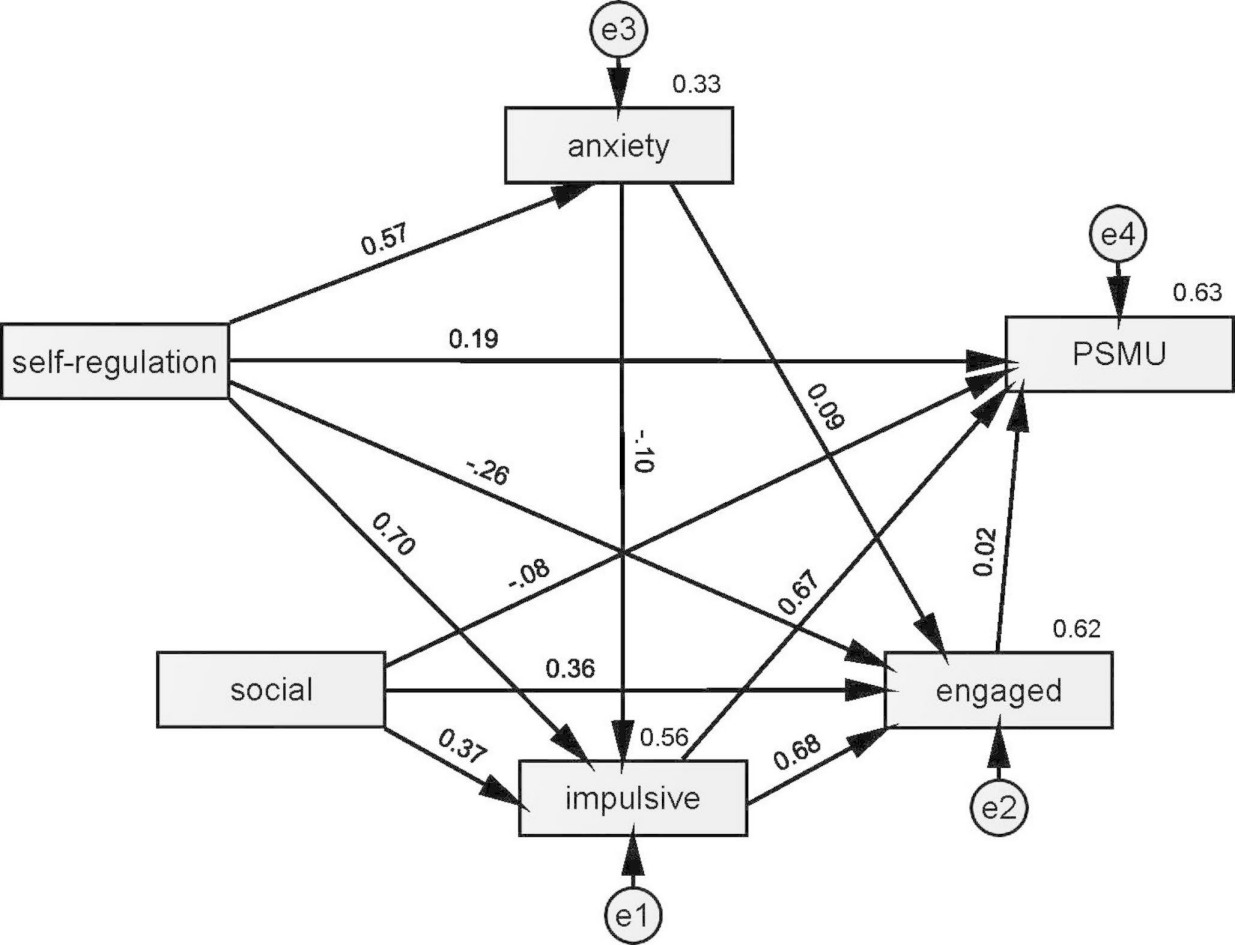
Structural model predicting PSMU (BSMAS) from attention dysregulation (ASRS), mediated by social media use motives (social, engaged, and impulsive), and anxiety (DASS_A)

An initial model test demonstrated poor fit (χ^2^/df = 100.087, RMSEA = 0.404, TLI = 0.256), improved by adding covariance between error terms for anxiety and depression (χ^2^/df = 6.451, RMSEA = 0.095, TLI = 0.959). This change was flagged in modification indices, and moreover these factors are highly correlated and derive from the same overall measurement tool (DASS-21). Despite this, we opted for a cleaner approach and simply deleted depression from the model. This was warranted as its only significant relationship was with attention dysregulation, and because of the implied redundancy with anxiety. The final model showed acceptable fit, albeit with some caveats: χ^2^/df = 4.708, and so is above the generally accepted threshold of 3, and RMSEA = 0.078, which is slightly high, but acceptable considering the “*p* close” test indicated a good fit (*p* = .102). Other fit indices were good: TLI = 0.971, CFI = 0.994, and CN = 336. With requisite caution due to validity issues described above, the obtained path model (see Fig. 2) provides insight into the relationships between attention dysregulation, social media motivations, and PSMU.

As shown in Table 3, and addressing our post-hoc research questions: **(1)** a significant positive association was observed between attention dysregulation (measured by ASRS) and PSMU (measured by BSMAS), mediated by impulsive use (revealed by the significant indirect effect). Engaged usage characteristics did not mediate between attention and PSMU; despite a strong significant negative direct effect between attention dysregulation and engaged use, there was no association between engaged usage and PSMU in this model (*β* = 0.022, *p* = .63). **(2)** Social-connection motivations showed a negative direct effect on PSMU, with a positive indirect/mediation effect on PSMU through impulsive use. No indirect effect was observed through engaged use. **(3)** Considering the role of anxiety in the model, attention dysregulation was a strong positive predictor (*β* = 0.574). While anxiety had weak negative relationship with impulsive use (*β* = − 0.103, *p* = .002), and positive with engaged use (*β* = 0.09, *p* = .003), the only mediation effect was through impulsive use, with a significant negative indirect effect from attention to PSMU through anxiety and impulsive use.

**Table 3:** Testing SEM Paths, Predicting PSMU (BSMAS) from Attention Dysregulation (ASRS), Mediated by Social Media Use Motives (Social, Engaged, and Impulsive), and Anxiety (DASS_A)

		Coefficients				95% Confidence Interval				
	Pathway		B	S.E.			Lower	Upper		*p*-value
1.	attention→PSMU		0.186	0.036			0.125	0.245		0.001
2.	attention→impulsive→PSMU		0.532	0.040			0.469	0.599		0.001
3.	attention→engaged		-0.264	0.042			-0.339	-0.198		0.001
4.	attention→engaged→PSMU		-0.007	0.012			-0.027	0.012		0.602
5.	social→PSMU		-0.076	0.033			-0.132	-0.024		0.015
6.	social→impulsive→PSMU		0.193	0.018			0.163	0.225		0.001
7.	social→engaged→PSMU		-0.001	0.011			-0.012	0.025		0.616
8.	attention→anxiety		0.574	0.027			0.53	0.617		0.001
9.	attention→anxiety→impulsive→PSMU		-0.045	0.014			-0.069	-0.022		0.001

These findings suggest that attention dysregulation predicts PSMU, mediated by impulsive social media use. In contrast, engaged usage motivations (using social media for the purpose of entertainment, or for information-seeking) do not appear to influence PSMU, either directly or as a path with attention dysregulation – however, attention dysregulation did have a direct negative effect on engaged use motives, which may indicate that users with issues regulating attention are less likely to use social media for purposes of seeking information or for fun/entertainment. Finally, the negative effect of social engagement motivations on PSMU may indicate that using social media for the purpose of social connection represents a generally productive or positive uses of the technology. That being said, there is a mediation effect from social use to PSMU through impulsive use. Finally, there was a multi-path mediation effect from attention to PSMU through anxiety and engagement. However, the effect of anxiety in the model is quite weak (*β* approximately − 0.10), and so emerges due to the substantial weights in other pathways. As such, this may be a spurious effect.

### Qualitative results

The second main research aim was to explore the experiences of social media use in university students who endorse indicators of attention dysregulation. Recall that the interview pool was selected based upon two criteria: (1) a clinical ASRS score of 5 or greater (which represents a positive screen for adult ADHD), and (2) a score within the highest or lowest quartiles on the BSMAS. This was intended to offer a range of PSMU experiences in the sample. Thematic analysis of interview transcripts and open-ended questionnaire responses lead to the identification of two themes and seven subthemes focused on issues of self-regulation and social media use motives. These themes and their prevalence in the sample are presented in Table 4.

**Table 4:** Prevalence of Themes as Total Number of Mentions, and Number of High and Low BSMAS Participants Who Mentioned Each Theme

Theme	Total No. of Mentions	No. of High BSMAS Participants	No. of Low BSMAS Participants
The Impossible Task			
A Conscious Effort	25	8	7
Getting Lost in Social Media	41	9	5
Out of Sight, Out of Mind	35	9	7
Purposeful Social Media Use			
Keeping Entertained	36	10	11
Staying Informed and Educated	36	9	6
Connecting with Others	29	9	9
Escaping Reality	35	10	10

Notably, while there was a slightly lower frequency of participants in the low BSMAS group who expressed difficulties managing their social media usage (especially “getting lost in social media”), the character of experiences appeared largely similar between high and low BSMAS categories, which raises some concerns about the effectiveness of the BSMAS in discriminating problematic social media use (at least within people who experience difficulties with attentional dysregulation). To exemplify this, Table 5 contrasts the experiences of participants across the spectrum of BSMAS scores, in view of two broad summative questions reflecting on the negative impacts of social media use, and whether this is perceived as balanced by the positives

**Table 5:** Examples of negative and positive impacts of Social Media Use Across BSMAS Scores

BSMAS score	Please describe negative impacts you experience from using social media	Do you feel that any negative impacts are balanced by the positives?
10	Constantly looking at or seeing other pretty females who have the “perfect” life and body, etc. Although it can all be an illusion it still impacts me negatively.	Not really.
12	When using social media I tend to lose track of time, which prevents me from doing more important things during the day.	Social media is a good place to see other people’s opinions on topics that you are interested, and when used positively, can create communities that help support each other.
12	Out of touch with reality. When you’re mindlessly scrolling for hours you get stuck and that becomes your reality.	In some way as social media can be quite informative.
24	Using Facebook to initially talk to or message friends about university work becomes an infinite scroll through the platform.	The negative is only balanced by the ability to communicate with friends that I’ve not seen in years.
27	Waste of time, don’t get anything done which leaves me feeling unaccomplished, lazy, depressed, upset.	Sometimes, but not as much. Like when I’m feeling overwhelmed or have a panic attack the only thing that helps is watching videos and photos of other people living their lives the way I wanted to or at least wish I could.
28	Some posts, especially news stories, cause immense stress and anxiety that can take over and disrupt the whole day.	Keeping in touch with long distance friends.

The question framing here (specifically asking participants to reflect on negative impacts) certainly has a role in generating the kind of examples seen. This was, however, prefaced by more neutral questions (e.g., “describe your engagement with social media in a typical day” and “are there aspects of your social media usage that you find especially problematic?”). Such questions did not reveal clear differences between experiences across BSMAS score categories either. One interview question (“Do you feel a strong urge to get onto social media?”) revealed consistent differences between high and low BSMAS participants (with low BSMAS participants tending to disagree, and high BSMAS in agreement). Given the general consistency across participants, it would not be productive to contrast BSMAS groups in thematic analysis presented below. Please note, individual participants are referred to with a random participant number (i.e., P1 through P24).

#### The “Impossible Task:” Self-Regulation of Social Media Use

Self-regulation of social media use, particularly cutting down on the amount of time spent on social media platforms was perceived by many to be very difficult: “withdrawing from social media use altogether felt like an impossible task” (P17). This difficulty in regulating social media use is explored in the following subthemes: (1) A Conscious Effort, (2) Getting Lost in Social Media, and (3) Out of Sight, Out of Mind.

##### A Conscious Effort

Regulating one’s usage of social media requires a level of effortful self-control that many do not believe they possess, expressed as “I don’t feel I have much of a capacity to self-regulate” (P23) and “I feel that I don’t have control over my attention or time management” (P16). Some have such difficulty, they require someone else to help with regulating their social media use, as one participant expressed: “I need someone else to kind of tell me, to just catch me out on that, because like once I’m on there and I’m scrolling, […] I’m stuck” (P7). Others describe their self-regulation as a “conscious effort” in which they focus on being “more mindful when browsing the internet and socials” (P17).

This conscious effort to regulate one’s social media use involves more than just an awareness when using social media. Many reflected on being aware whilst using social media for longer periods than intended: “I unknowingly refresh to see more even after I told myself to set a time to get off or continuously scrolling past knowing I’m wasting time” (P14). Some described setting timers as a reminder to stop using social media, but “it’s just very easy […] to just press ignore” (P7). Awareness of problematic use is not sufficient for self-regulation as “motivation” and “self-control” also play a large role: “I have no self-regulation in regards to social media, I do try to maintain usage but it usually doesn’t work” (P13).

##### Getting Lost in Social Media

Some participants expressed being aware of their overuse whilst on social media, yet others articulated that they “get lost in [their] usage of social media” (P21). One participant went as far as to describe it as a “trance”-like state that they need to “snap […] out of” (P14). Unconsciously checking social media out of habit and losing track of time when on social media are key aspects of mindless use. P7 expressed that:

[checking social media is] 100% an automatic thing. I would just like go to bed, lay down and just immediately go on my phone and start scrolling, and before I realise it, like four hours later, then I become conscious.

The experience of losing track of time on social media is common, with many describing similar occurrences: “Instead of replying to the one message sent, I keep scrolling through and what initially should have been a one minute interaction becomes 30 minutes of endless scrolling” (P10). Another communicated that “time seems to move quickly” (P24) when they are using social media and that this results in excessive use. Similarly, unconscious habitual checking was a typical experience among participants, as many expressed “going on social media apps without realising and mindlessly scrolling due to habit” (P16).

##### “Out of Sight, Out of Mind”

The act of “subconsciously just [picking] it up and […] scrolling” (P4) and being unable to “resist looking at [their] phone” (P21) when it is easily accessible is why many take an “out of sight, out of mind” (P14) approach to self-regulation of social media. One participant expressed that they are “easily distracted from university study or tasks that require large amounts of concentration” and that this is exacerbated by social media: “the fact that social media is so readily around me through my phone being on me at all times [and] my own inability to regulate my attention has caused the overuse” (P18). As social media is a readily available distraction, the key strategy of self-regulation used by participants was to reduce the accessibility of social media. For some, this strategy involved moving social media out of reach, trying to “simply just hide it and forget about it for the rest of the day” (P13).

Removing social media from their immediate area has been found to be a successful self- regulation technique, as P14 expressed:

Having my phone out of sight and out of reach from me has proved to be effective and I think it’s true the saying, “out of sight, out of mind.” Setting alarms wasn’t as effective because I would usually just snooze or just stop the alarm and keep scrolling.

Similarly, others described “planning out [their] day” and keeping busy as their strategy for “staying distracted” from social media (P8). That is, when asked about their self-regulation of social media use, some participants voiced that they are able to stop using social media when there is “something [they are] invested in,” such that they have “no urge to pick up [their] phone” when they are engaged in another effortful activity (P5).

A common self-regulation strategy described by participants was to remove their access to social media entirely. For many, this meant deleting social media applications: “I deleted a few social media apps so as to reduce the amount of time I spend on them” (P15). Others describe doing a “social media cleanse” or going on a holiday with no reception as a means of self-regulating social media use. Less drastic than this all-or-nothing method were those that muted notifications to reduce distractions or caused social media platforms to be “a little more difficult to access through using them on a laptop” instead of a smartphone as they believed “completely removing social media will somehow lead to a relapse” (P15). Reducing their access and complicating the process of checking social media is the most common strategy used to aid in self-regulation, as expressed by P11: “I have deleted most platforms of social media in order to reduce daily consumption and hidden away the apps that I do currently have to avoid using it mindlessly.”

#### Purposeful Social Media Use

While many reported using social media mindlessly, almost all participants also described using social media for a variety of purposes. These intentional uses of social media are explored in the following subthemes: (1) Keeping Entertained, (2) Staying Informed and Educated, (3) Getting Motivated, (4) Connecting with Others, and (5) Escaping Reality.

##### Keeping Entertained

Social media use as a form of entertainment was commonly reported, with many responding that “entertainment” (P16) was their primary goal when using social media. Extending beyond entertainment purposes, social media is used to fill any spare time that individuals may have: “the purpose I have for using social media is to keep me entertained when I have nothing else to do” (P9). The phrase “nothing else to do” (P2) was repeated by a number of participants who describe using social media as a way of passing time or combating boredom: “I just scroll until I find an interesting video to waste time” (P10). In contrast, social media is also used to cultivate new hobbies and “introduce […] new areas of interest” (P19).

##### Staying Informed and Educated

Social media is a source of information and education for many individuals: “I more so use it to educate myself and learn up on the types of things I’ve been wanting to teach myself” (P17). Notably, many participants expressed that social media was their primary method of staying “up to date with social issues” (P9) and current world events. Others described social media use as a tool that complements their university studies, as they use social media platforms such as YouTube as “a learning tool for difficult concepts that [they were] not able to understand at university” (P10). Searching for “another perspective” (P10) proves to be valuable as social media as broadening their world view, as expressed by P19:

It has also introduced me to new areas of interest, provided information and kept me informed. It reveals to us that the world is vastly more diverse, interesting and complex than my world-view.

From remaining informed about current events to educating oneself about new topics, purposeful use of social media for some includes seeking information and education.

##### Connecting with Others

More prevalent than seeking motivation was the purposeful use of social media to interact with friends and family. All participants voiced that a key purpose of their social media use was to connect with others: “social media is a way to connect with and keep up with loved ones, […] reconnect with friends that I thought I had lost” (P19). Particularly salient was social media as a “way to stay in contact with friends and family overseas” (P24) and “the ability to communicate with friends that I’ve not seen in years” (P10). Connecting with others, specifically to “talk to people and see what they are up to” (P11) through posted content, was found to be the core use of social media for most individuals.

##### Escaping Reality

Lastly, a common use of social media is as “a coping mechanism to escape and deal with a different sort of ‘reality’” (P17). For many, social media use is a means of procrastinating and distracting oneself from tasks: “I’m a really big procrastinator and social media is a way I can escape doing my work until the last minute” (P9). Social media as an escape was a common experience as many expressed using social media for the purpose of distracting themselves and delaying confronting any issues they may be experiencing. One participant poetically described social media as “providing temporary simple pleasure that has the ability to distract an individual from the world” (P12). The escapism is not limited just to avoidance of pending tasks and to “explore the lives of others instead of focusing on [one’s] own” (P10). Many individuals expressed that social media use was a means of “filling an emotional void” (P16) and seeking “temporary alleviation of […] anxieties” (P19). While some of this is evocative of problematic use, we note that this kind of motivation can still be adaptive, as a way for individuals to gain distance from an emotion or problem that could otherwise become overwhelming.

## Discussion

The present study investigated the relationships between attention dysregulation, social media use motives, psychological wellbeing factors (anxiety and depression) and PSMU in a sample of Australian university students. A nested mixed-methods design was used to first identify the relationship of variables through SEM analysis, and then gain insight from interview or open-ended questionnaire within a subset of participants who demonstrated indicators of attention dysregulation (meeting screen threshold for adult ADHD), recruited with a spread of high and low BSMAS scores. Our SEM principally indicated that scores on the ASRS (reflecting attentional/self-regulation) positively predict scores on the BSMAS (reflecting PSMU/SMA) through usage characteristics of procrastination and unintentional/habitual use (we categorise as impulsive social media use). There was also an indication that social-connection motivations negatively predict BSMAS scores. There were no clear effects of wellbeing factors (anxiety or depression) in the model, the only convincing associations being that attentional dysregulation predicts elevated anxiety and depression.

Prior research has demonstrated the relationship between attention dysregulation and PSMU (Boer et al., 2020; Ko et al., 2009; Reinecke et al., 2022) have presented a detailed conceptual analysis of the complex role of self-regulation, alongside various contextual and motivational factors. While the model generated in this study found a particularly strong effect for impulsive use, it is likely that common (self-regulation) processes drive scores on both the ASRS as well as impulsive use. Given that the impulsive use items pertain more directly to social media use behaviours, it makes sense then that the relationship with BSMAS was particularly strong. From this, we argue that the BSMAS is flawed by its lack of a defined self-regulation component, and further by its insensitivity to contextual factors that define individual subjective experiences of social media use as problematic or otherwise (however, this is more of an issue with how such measures are used in research, rather than with the tools directly). These themes were reflected particularly clearly in the qualitative data, where a common narrative expressed substantial challenges associated with self-regulating social media use, the distress of such experiences, as well as problematic use tending to be balanced by affordances of social connection.

While there are elements of the BSMAS that tap into self-regulation, most notably *mood modification* (“Used social media in order to forget about personal problems”) and *relapse* (“Tried to cut down on the use of social media without success”), these lack conceptual clarity. This is particularly prominent for *mood modification*. The underlying idea is that using social media for the purpose of forgetting about personal problems is maladaptive, as articulated by Andreassen (2015, p.179), because “SNS addicts engage in social networking to gain control, but become controlled by their social networks.” There is an internal logic to this assumption, reinforced by the cluster of behaviours otherwise reflected in the addiction framework (uncontrollable urges, failure to restrict use, and conflict), which may look a lot like addiction (Anderson & Wood, n.d.). However, whether mood modification behaviours are adaptive or maladaptive will depend largely on the context in which they occur, potentially providing necessary relief from unavoidable or unchangeable internal or external stimuli, as well as access to information and social connection. While there would surely be some contexts in which the BSMAS (and other measures of PSMU/SMA) validly indicate a problematic relationship with social media, it is absolutely necessary to view such measures as broad screening tools rather than concrete measures of a particular phenomenon – especially when used in the context of broad survey studies of the general population where such screening measures are arguably prone to false positives (Maraz et al., 2015). This critique naturally applies also to our use of the ASRS, and despite clear indications that participants selected from it displayed indications of difficulties with self-regulation, it is nevertheless a limitation in our research. That being said, we are not endeavouring to promote claims for associations between ADHD and SMA.

More critically, researchers have questioned the prevalence and severity of social media addiction (Carbonell & Panova, 2017), and the concept of addiction in other areas of technology use (Aarseth et al., 2017; Ellis, 2019; Przybylski et al., 2017). This has led to a growing acknowledgement that the construct lacks a firm grounding, with Carbonell & Panova (p.48) cautioning that “although similarities between excessive use of SNS’ and addiction may exist, the pathologizing of the new computer-mediated form of communication needs to be met with a cautious and critical eye”. They reason that the context of use makes all the difference in terms of understanding social media use, and behaviours that may be captured in a measure of social media addiction (like conflict, salience, and mood modification) may be better understood in terms of normal psychological or developmental processes. For instance, a young person may prioritise engagement with social media over attending to classwork due to that action furthering their social capital development, and not because they are experiencing or at risk of social media addiction. Other researchers, such as Billieux et al. (2018) and Meier (2022) similarly argue that focus must shift from studying “problematic use”, to studying specific problems and processes that are most meaningful to users of social media.

One such meaningful problem is how self-control strategies can be best employed, in what contexts, to manage various aspects of behaviour related to social media use. For example, Meier (2020) reported a diary study investigating the link between mobile checking habits and procrastination, framing the research as investigating a “key functional problem” by “predicting problematic outcome (i.e., procrastination) through key aspects of person- and day-level mobile connectivity (i.e., checking habits)—rather than assuming mobile media to be problematic per se” (Meier, p.273).

In relation to this topic, our thematic analysis found that awareness of problematic use and intentions to reduce or halt social media use are not sufficient to lead to self-regulation – indeed, self-regulation of social media use was unsurprisingly perceived as effortful and difficult. The individuals who participated in our interview research reported some success when endeavouring to manage social media use by disrupting their access to social media through situational self-control strategies. Typically, this was done by (temporarily) uninstalling social media applications, deactivating social media accounts, or placing the device used to access social media in a different location. Such strategies are reportedly more effective than reactive or sheer willpower approaches, but simultaneously require more intentional effort (Brevers & Turel, 2019). Indeed, self-regulation strategies were generally not met with lasting success in terms of an ongoing sense of effective management of social media use, likely driven by participants’ underlying difficulties with self-regulation. Further research is needed to understand the processes involved, and to explore methods that best support individuals (especially those most prone to self-regulatory failures) in managing their social media use.

## Research Limitations

We have discussed some of these core limitations above, but it is worth reiterating that there are major limitations within the PSMU/SMA field broadly, and that these have implications for the present research. Carbonell and Panova (2017), for instance, argue that addiction is a flawed model for framing social media use behaviours, as there is little compelling research identifying the addiction construct, or the severity of effects. They also argue that there is poor alignment between given measures of addiction and the behaviours or experiences they are purported to measure. This is echoed in debates within the areas of internet gaming (Aarseth et al., 2017; Przybylski et al., 2017) and smartphone use fields (Ellis, 2019; Griffiths et al., 2017) respond that gaming addiction (which would extend to other technology use problems) is a syndrome, characterized by a set of associated symptoms that tend to occur under specific circumstances, and therefore resist consistent description and symptomatology. While we should be hesitant about applying the terminology of addiction (or even softer phrasing such as “addiction-like”*)*, we agree with Griffiths et al. that individuals nonetheless experience substantial distress in relation to their technology use. Indeed, from the qualitative data of the present study, it was clear that individuals do experience substantial distress in relation to their social media use. However, much more care is needed in appropriately framing social media research, and the conclusions drawn therefrom.

We also note that despite strengths in the nested mixed-methods design, the research is limited as participants were drawn from a pool of first-year university psychology students. The sample is meaningfully representative of the kinds of people who struggle with social media usage, but does not necessarily generalise beyond the context of young people who attend university. It is also noteworthy that in both quantitative and qualitative samples, 80% of participants were women. Finally, it must be noted that the COVID-19 pandemic may have influenced experiences around social media use in the participants of this research. During data collection, Sydney experienced a drastic increase in COVID-19 cases and was placed into a lockdown. A number of participants mentioned the impact of COVID-19 and isolating within homes in their responses in interviews and open-ended questionnaires, stating that social media use was increasingly important for entertainment and connection as they had more individual time and that it was one of few methods of staying in contact with others.

## Conclusions

We agree that social media, as with use of many other new technologies, is overpathologized (Billieux et al., 2015). However, many people do experience their SMU as problematic, complex, and difficult to manage. We believe that our findings offer some useful points for further development. First, the BSMAS may be improved by the addition of an explicit self-regulation component. Second, it requires further evaluation in the context of capacities for attentional functioning. The interview data demonstrated that individuals selected for attentional dysregulation describe very similar kinds of experiences with SMU, despite being split into groups that should differ in the intensity with which they experience their use of social media as problematic. This either suggests that the BSMAS is not very good at distinguishing such experiences in general, or that it lacks effectiveness in the case of people with attentional dysregulation. Thirdly, the identification of a mediating role of impulsive use within the problematic use construct contributes to understanding how it develops, and may help explain why the interview participants express generally problematic experiences with social media use regardless of score on the BSMAS. Finally, the interview data provides a rich understanding of how individuals exhibiting attentional dysregulation manage (and struggle with) their social media usage, indicating that situational strategies may be particularly useful for this goal, yet are difficult to enact, especially for individuals with low trait self-control.

While the present research was developed from within a PSMU framework, we nevertheless hope that it contributes to the growing voices urging care with the way social media research is formulated and discussed. Important questions remain that are deserving of ongoing research efforts, and which need to be appropriately contextualised in the behaviours, processes, and outcomes that matter.

## Data Availability

The datasets generated and analysed in the current study are stored in perpetuity according to the Western Sydney University Human Research Ethics Committee Extended Consent guidelines, meaning that data is available for use in related research that meets requirements of ethics review. Researchers who wish to access the data may do so by contacting the lead author, or the WSU HREC if the author is uncontactable (HREC approval number H14268).

## Appendix A

### Open-ended questionnaire/interview protocol

(all items presented in Qualtrics, with free response text boxes)

Please describe (briefly) your engagement with social media in a typical day.

Consider the following (you don’t need to answer all questions, they are listed here as prompts for you to respond if they resonate with you):

- Do you use social media for set times, randomly, when bored?
- Do you usually have a goal/purpose for using social media?
- Do you lose track of time when on social media?
- Any other/different points you feel are relevant?

Are there aspects of your social media usage that you find especially problematic?

- This could include things like excessive use, frequently losing track of time, impacting your work or study, causing stress, anxiety etc.)

Please describe what this problematic use is like for you:

- Do you often feel a strong urge to get onto social media (if yes, how does this impact your day-to-day life)?
- Do you often use social media as a distraction, or to forget about difficult emotions or problems (if yes, how does this impact your day-to-day life)?
- Any other/different points you feel are relevant?

Please describe the negative impacts you experience from using social media (if any):

- If relevant, do you feel that any negative impacts are balanced by the positives of social media for you (please describe)?

A key aim of this research is to explore how individual capacity for “self-regulation” might impact their experiences around social media usage (specifically, aspects of usage that they feel have a negative impact).

In answering the next set of questions, consider the following definition:

Attentional regulation is the capacity to choose where and how we focus our attention, which enables us to plan and carry out our day-to-day activities and manage distractions….

Do you feel that your capacity for self-regulation has some impact on the way you use social media (please describe)?

Do you experience specific areas of difficulty with managing social media use? Consider, for example, issues with the following:

- Managing impulsive thoughts/behaviours?
- Managing excessive use?
- Halting social media once you’ve started?
- Repetitive refresh/endless scrolling?
- Anything else you’d like to describe?

Do you feel that it is necessary for you to cut down on your social media use (if yes, please describe why)?

Have you previously attempted to cut down on your social media use?

Please describe some of the methods you’ve used to attempt this?

How successful were these attempts (can you identify why these attempts were, or were not, successful)?

Can you describe how you felt/feel when restricting access to social media?

Anything else you’d like to describe?

## Appendix B

### Exploratory Factor Analysis

An initial eight factors were extracted using Maximum Likelihood method, based on Eigenvalue > 1, with Promax rotation (Kappa = 4), accounting for a cumulative variance of 54.5%. The data showed good factorisability, with KMO = 0.943, and majority of extraction communalities > 0.5 (and very few < 0.3). The resulting pattern matrix somewhat confirmed expectations, but with notable exceptions.

Firstly, DASS stress scale items loaded on a single factor with DASS anxiety items (perhaps reflecting general reactivity), with much weaker loadings for the stress scale items. Second, DASS depression scale item 2 did not behave as expected, instead loading with ASRS items 1–4. This is understandable as this item assesses capacity to initiate intentional behaviour (i.e., “I found it difficult to work up the initiative to do things”), thus overlapping with the ASRS executive functioning construct. Third, items 5 and 6 of the ASRS exhibited poor loadings (low values, and in separate factors), likely because these items relate to the behavioural component of ADHD (e.g., “feeling compelled to do things, like you were driven by a motor”), compared with items 1–4, which relate to executive functioning component (e.g., “How often do you have difficulty getting things in order when you have a task that requires organisation?”).

Regarding the social media motivation items, entertainment (e.g., “I use social media because it is fun”) and information seeking (“I use social media to find information”) loaded as a factor together, along with one stress relief motivation item (“I use social media to recover from work or other exhausting tasks”), perhaps indicating a general purposeful or engaged usage orientation. This was distinct from social-connection motivations (“I use social media to catch up with friends”), which loaded together on a separate factor. Also distinct were items indicating usage that is characterised as automatic or habitual, and for procrastination (“I check my social media without thinking about it”, “I use social media even though I have more important things to do”), which loaded on a factor together. This is taken to indicate a general impulsive or reduced control component.

Lastly, it is notable that BSMAS item 3 (mood modification; “used social media in order to forget about personal problems”) had lower loading (0.479) in comparison with the rest of the BSMAS (next lowest loading = 0.615). This item is problematic as it conceptually overlaps with the DASS stress scale, and stress relief motivations (i.e., all related to processes of mood modification). It is also a problem item because using social media to shift one’s mood, by distracting oneself from personal problems, can be a rational, adaptive, and even constructive coping response and so may not well capture the construct.

As such, a second run (removing BSMAS item 3, DASS stress scale, DASS depression scale item 2, and ASRS items 5 and 6) resulted in seven factors extracted, accounting for a cumulative variance of 55.8% and sufficient factorisability (KMO = 0.921). The pattern matrix (see Table B.1) confirms factor structure for adjusted BSMAS, DASS anxiety and depression, and somewhat for ASRS as well (albeit loadings are < 0.70). The usage motivation groupings identified previously continue to hold, however with some loading issues. Specifically, one stress usage item has low loading at 0.302, and so was removed; stress item 2 displays some cross-loading with the impulsive/control factor, but as this cross-loading is very close to 0.3 the item was retained as part of the purposeful usage factor; information item 2 has lower loading at 0.355, and so was also deleted). It is noteworthy that impulsive social media usage (i.e., habitual use and procrastination) emerged as a distinct independent factor, suggesting that this reflects a construct distinct from that represented by the BSMAS.

**Table B.1:** Exploratory Factor Analysis Pattern Matrix (items in bold were excluded when moving to CFA/SEM)

	Factor						
	1	2	3	4	5	6	7
BSMAS_1			0.619				
BSMAS_2			0.640				
BSMAS_4			0.739				
BSMAS_5			0.633				
BSMAS_6			0.572				
MOT_AUT1				0.784			
MOT_AUT2				0.717			
MOT_PRC1				0.762			
MOT_PRC2				0.676			
MOT_STR1					0.**302**		
MOT_STR2				0.**308**	0.421		
MOT_INF1					0.404		
MOT_INF2					0.**355**		
MOT_FUN1					0.816		
MOT_FUN2					0.867		
MOT_SOC1							0.683
MOT_SOC2							0.949
DASS_A1	0.477						
DASS_A2	0.860						
DASS_A3	0.807						
DASS_A4	0.613						
DASS_A5	0.685						
DASS_A6	0.869						
DASS_A7	0.663						
DASS_D1		0.705					
DASS_D3		0.825					
DASS_D4		0.628					
DASS_D5		0.656					
DASS_D6		0.860					
DASS_D7		0.936					
ASRS_1						0.698	
ASRS_2						0.552	
ASRS_3						0.548	
ASRS_4						0.475	

Extraction Method: Maximum Likelihood

Rotation Method: Promax with Kaiser Normalization

a. Rotation converged in 8 iterations

## Appendix C

A marker variable for screening CMV comprised items from the ‘describing’ sub-scale of the Five Factor Mindfulness Questionnaire (Baer et al., 2006), which refers to the ability to label internal experiences with words (participants completed the FFMQ as part of their participation in a broader study). Whilst the attention and emotion regulation components of mindfulness do overlap with study variables, and there may be some relationship between psychological wellbeing and the ability to verbalise experiences, the theoretical overlap is more obscure, and does not clearly relate to social media usage or with the normal bounds of attentional function. Nevertheless, in an effort to make the marker variable more neutral, we removed items from the describing scale that relate to emotional content (keeping, for example, “I can easily put my beliefs, opinions, and expectations into words” and removing “Even when I’m feeling terribly upset, I can find a way to put it into words”). Following recommendations of Simmering et al. (2015), this is a reasonable marker as it has minimal theoretical overlap with the other variables in the model, while the methods of data collection (Likert-type scales completed in the same testing session) should elicit similar response processes and tendencies. Further CFA results showed generally low associations between the marker and all study variables, with highest weightings around − 0.3 for attention and depression, otherwise generally much lower than other weightings in the model.
